# 3D-printed scaffold-based strategies for enthesis regeneration: a systematic review of the literature

**DOI:** 10.3389/fbioe.2026.1876882

**Published:** 2026-08-31

**Authors:** Marco Minelli, Luca Bertolino, Vincenzo Longobardi, Simone Micalizzi, Federica Potere, Giuseppe Anzillotti, Tommaso Bonanzinga, Elizaveta Kon, Federico Della Rocca, Paolo Oliva

**Affiliations:** 1 Department of Biomedical Sciences, Humanitas University, Pieve Emanuele/Milan, Italy; 2 IRCCS Humanitas Research Hospital, Rozzano/Milan, Italy; 3 Humanitas University, Pieve Emanuele/Milan, Italy

**Keywords:** 3D-printing, bone, enthesis, regeneration, scaffolds, tendon

## Abstract

**Background:**

The enthesis is a specialized transitional tissue that enables load transfer between tendon and bone through a graded tendon–fibrocartilage–bone interface. Following injury or surgical repair, regeneration of the native enthesis remains challenging, often resulting in mechanically inferior fibrous scar tissue. Additively manufactured three-dimensional (3D) scaffolds have emerged as a promising strategy to recreate the structural and biological complexity of the tendon–bone interface. This systematic review evaluated the current preclinical evidence on 3D scaffold-based approaches for enthesis regeneration.

**Methods:**

This systematic review was conducted according to PRISMA 2020 guidelines and registered in PROSPERO (CRD420261388606). PubMed, Embase, and Web of Science were searched from inception to 13 February 2026. Original preclinical studies investigating additively manufactured 3D scaffold-based strategies for tendon–bone interface or enthesis regeneration were included. Data regarding scaffold design, biological augmentation, and *in vitro*, *in vivo*, and biomechanical outcomes were qualitatively synthesized. Methodological quality was assessed using SYRCLE’s Risk of Bias tool.

**Results:**

Nine preclinical animal studies published between 2019 and 2025 met the inclusion criteria. Most studies used rabbit rotator cuff repair models. Polymer-based scaffolds, particularly polycaprolactone and poly (lactic-co-glycolic acid), were the most frequently used materials. Advanced architectures included gradient, multiphasic, bioprinted, and coaxial constructs, often combined with mesenchymal stem cells, growth factors, or controlled-release systems. Across studies, scaffold-based strategies consistently promoted enhanced lineage-specific differentiation, fibrocartilage formation, collagen organization, and interface maturation compared with controls. Biomechanical testing demonstrated improved ultimate load, stiffness, tensile strength, or energy absorption in all experimental groups. Constructs combining biomimetic architecture with biological augmentation generally achieved good outcomes, although regenerated interfaces remained inferior to native tissue. Risk-of-bias assessment showed overall unclear methodological quality due to insufficient reporting.

**Conclusion:**

Additively manufactured 3D scaffold-based strategies show promise for improving enthesis regeneration in preclinical models. Constructs integrating spatially organized architecture with controlled biological signaling demonstrated the most favorable structural and biomechanical outcomes. However, current evidence is limited to heterogeneous short-term animal studies, and complete restoration of native enthesis structure and function has not yet been achieved.

## Introduction

The enthesis, or tendon–bone interface, is a specialized transitional tissue that enables efficient load transfer between compliant soft tissue and rigid bone (Shaw, 2026; Tits et al., 2021; Killian, 2022). In fibrocartilaginous entheses, this transition is typically organized into four zones—tendon, unmineralized fibrocartilage, mineralized fibrocartilage, and bone—whose gradients in structure and mechanical properties are essential for physiological function (Shaw, 2026; Tits et al., 2021; Killian, 2022; Deymier et al., 2017; Golman et al., 2021). Disruption of this interface is a major problem in musculoskeletal injuries and reconstructive procedures because the native insertion is difficult to recreate once damaged (Huang et al., 2026; Atesok et al., 2014). This is relevant because procedures such as rotator cuff repair are among the most frequently performed orthopaedic interventions (Herzog et al., 2018; Jain and Khazzam, 2024).

Despite advances in surgical fixation and repair techniques, healing at the tendon–bone interface remains suboptimal. Rather than regenerating the native enthesis, postoperative healing often results in scar-like fibrous tissue with inferior organization and biomechanical performance, contributing to failed integration and potential re-injury (Kanazawa et al., 2016; Zhang et al., 2023). These biological and mechanical limitations have driven growing interest in regenerative strategies that aim to better replicate the hierarchical architecture and microenvironment of the native interface.

Among these strategies, three-dimensional scaffold-based approaches have emerged as a particularly promising platform for enthesis regeneration. 3D scaffolds can be engineered to provide spatially controlled structural and biological cues that mimic the native tendon–fibrocartilage–bone transition (Huang et al., 2026; Zhang et al., 2023; Zhu et al., 2018). Recent work has explored a wide range of constructs, including aligned nanofibrous scaffolds, multilayered or gradient scaffolds, 3D-printed and bioprinted architectures, mineralized composites, and decellularized matrix-based designs, often combined with cells, growth factors, or other bioactive agents (Sensini et al., 2021; Govindaraju et al., 2023; Zhu et al., 2021; Xie et al., 2025). Experimental studies suggest that such scaffolds may improve fibrocartilage formation and biomechanical integration at the repair site (Sensini et al., 2021; Govindaraju et al., 2023; Zhu et al., 2021; Xie et al., 2025).

However, the available evidence is dispersed across different anatomical models, scaffold platforms, and outcome domains. In this scenario, a dedicated synthesis of original studies on additively manufactured 3D scaffold-based approaches for enthesis or tendon–bone interface regeneration is still needed. Therefore, the aim of this systematic review is to evaluate original research on 3D scaffold-based strategies for enthesis or tendon–bone interface regeneration, with particular attention to scaffold characteristics, biological augmentation, and structural, histological, and biomechanical outcomes.

## Materials and methods

### Database search

**FIGURE 1:**
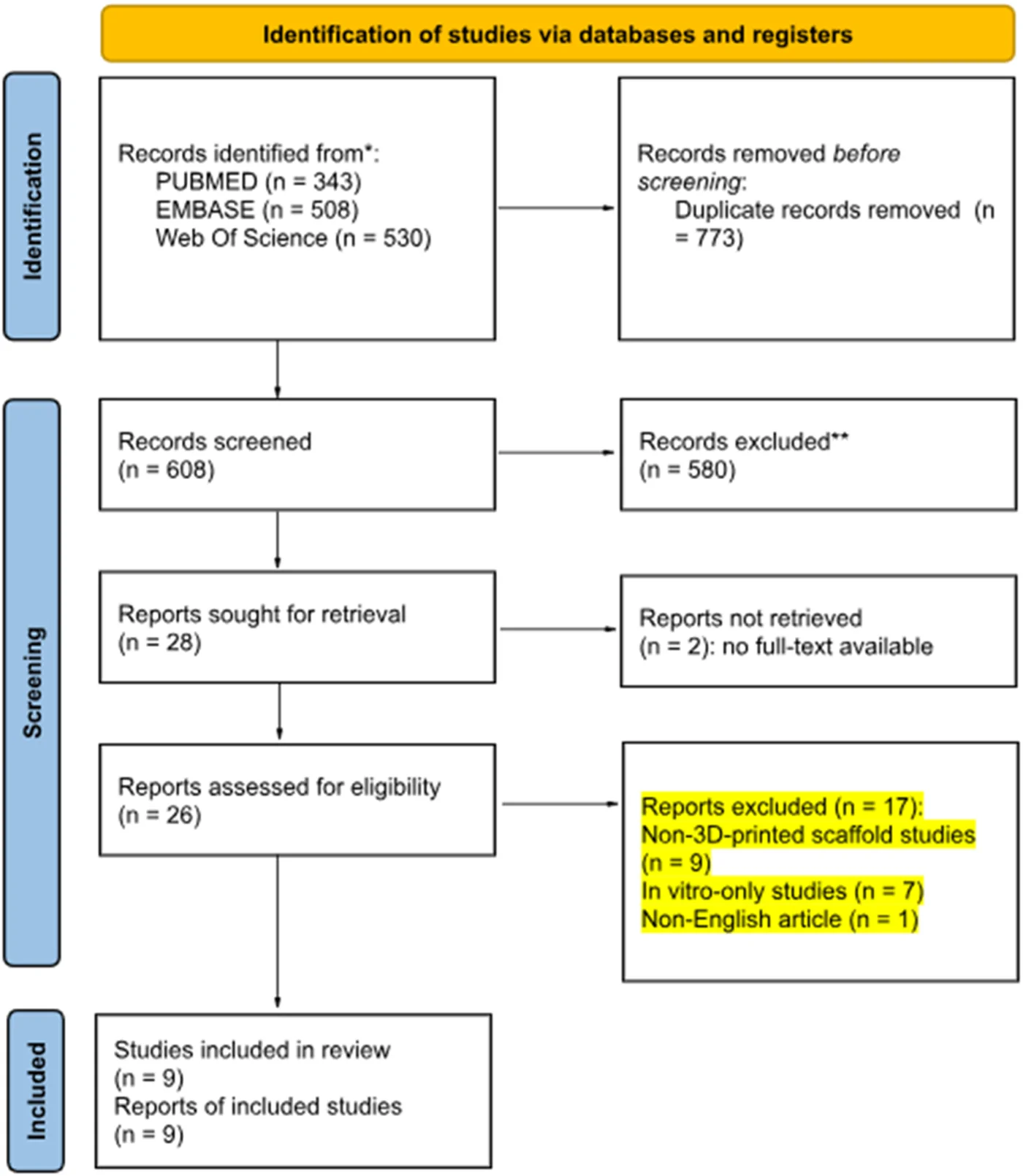
Flowchart of the selection process according to the PRISMA 2020 (Preferred Reporting Items for Systematic Reviews and Meta-Analyses) guidelines.

This systematic review was conducted in accordance with the Preferred Reporting Items for Systematic Reviews and Meta-Analyses (PRISMA) 2020 statement, as illustrated in Figure 1. The review protocol was registered in PROSPERO on 06-05-2026 (registration number [CRD420261388606]). A systematic literature search was performed in PubMed, Embase, and Web of Science from database inception to 13 February 2026. The following search strategy was used (“enthesis” OR “tendon-bone interface” OR “bone-tendon interface” OR “osteotendinous junction”) AND (“scaffold” OR “scaffolds” OR “biomaterial” OR “biomaterials” OR “tissue engineering” OR “3D printing” OR “bioprinting” OR “additive manufacturing” OR “multiphasic” OR “gradient” OR “functionally graded”) AND (“healing” OR “regeneration” OR “repair” OR “fibrocartilage” OR “histology” OR “histological” OR “osteogenesis” OR “chondrogenesis” OR “tenogenesis” OR “biomechanical” OR “mechanical testing” OR “ultimate load” OR “stiffness” OR “failure stress” OR “energy to failure” OR “pull-out test” OR “tensile test”). In addition, the reference lists of all included studies were manually screened to identify any potentially relevant articles not retrieved through the electronic database search. The full search strategies were developed separately for each database according to its specific syntax and indexing system, and are reported in Supplementary Table S1.

### Study selection

**FIGURE 2:**
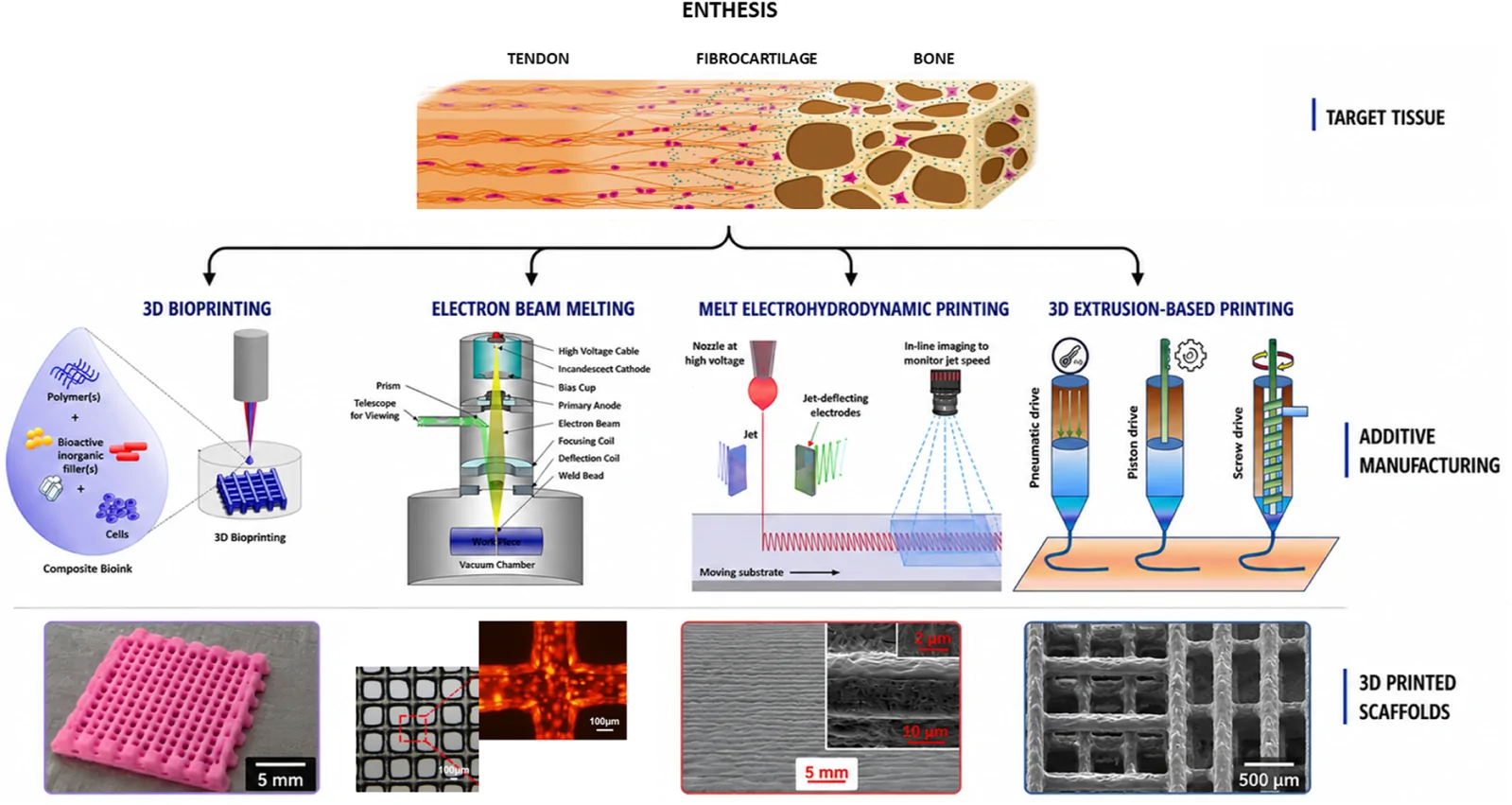
Schematic representation of the native tendon-fibrocartilage-bone enthesis and the main additive manufacturing technologies currently investigated for enthesis regeneration. These enable the fabrication of biomimetic scaffolds with tailored architecture and biological functionality to recreate the graded tendon-bone interface. Representative scaffolds produced by each technique are shown in the lower panel.

Studies were considered eligible if they were original research articles investigating scaffold-based strategies for enthesis or tendon–bone interface regeneration. Eligible studies had to evaluate three-dimensional scaffold constructs in which the main scaffold architecture was generated through an additive manufacturing process, including extrusion-based 3D printing, bioprinting, melt electrohydrodynamic printing, coaxial electrohydrodynamic printing, or electron beam melting. A schematic representation of the evaluated studies is reportend in Figure 2. For the purpose of this review, a “3D-printed scaffold” was therefore defined as a construct whose geometry and spatial organization were primarily established through layer-by-layer or additive fabrication. Studies in which the scaffold was mainly fabricated using non-additive approaches, such as electrospinning, solvent casting, freeze-drying, or decellularization alone, were excluded, even if a 3D-printed element was secondarily present. Studies also had to employ preclinical *in vivo* animal models of tendon–bone interface and to assess scaffold- or biomaterial-based constructs specifically designed to promote tendon–bone interface healing by reporting at least one relevant outcome, including *in vitro*, *in vivo*, or biomechanical findings. Articles were also excluded if they did not report any relevant *in vitro*, *in vivo*, or biomechanical outcomes. All search results were imported into Rayyan (Cambridge, MA, United States) to facilitate duplicate removal and screening. After duplicates were removed, two independent reviewers (M.M and L.B.) screened titles and abstracts according to the predefined eligibility criteria. Full texts of potentially relevant studies were then retrieved and independently assessed by the same reviewers. Any disagreements were resolved through discussion and consensus. The reference lists of selected studies were also examined to identify additional eligible articles that may have been missed during the original search. Furthermore, when multiple publications originated from the same study group, sample characteristics and reported data were compared to avoid duplication of extracted data.

### Data extraction and synthesis

Two reviewers (G.A. and F.P.) independently examined the full texts of the included studies and extracted the relevant data using a standardized Microsoft Excel spreadsheet (Microsoft Corporation, Redmond, WA, United States). The extracted information included study characteristics, experimental model, anatomical site, injury or reconstruction model, scaffold type, fabrication method, scaffold composition, structural design, biological augmentation, comparator groups, follow-up duration, and the main *in vitro*, *in vivo*, histological, radiological, biological, and biomechanical outcomes reported in each study. Biomechanical outcomes of interest included, where available, ultimate load, stiffness, failure stress, energy to failure, and failure mode or failure site. Given the expected heterogeneity in scaffold composition, fabrication strategy, animal models, follow-up periods, and reported outcome measures, the findings were synthesized qualitatively through a structured narrative approach.

### Risk of bias assessment

The methodological quality of the included studies was assessed using SYRCLE’s Risk of Bias tool for animal studies. Two reviewers (V.L. and S.M.) independently performed the risk-of-bias assessment, and any disagreements were resolved through discussion and consensus.

## Results

### Study selection

A total of 9 studies met the predefined eligibility criteria and were included in the qualitative synthesis (Zhang et al., 2023; Kim et al., 2024; Ni et al., 2025; Zhu et al., 2025; Bai et al., 2025; Chen et al., 2020; Chen et al., 2019; Bai et al., 2024; Bai et al., 2022). All included articles were preclinical animal studies and were classified as level V evidence.

### Study characteristics

The included studies were published between 2019 and 2025. Eight of the 9 studies investigated tendon–bone interface regeneration in a rotator cuff model involving the supraspinatus tendon (Zhang et al., 2023; Kim et al., 2024; Ni et al., 2025; Bai et al., 2025; Chen et al., 2020; Chen et al., 2019; Bai et al., 2024; Bai et al., 2022), whereas 1 study evaluated tendon–bone healing in a patellar ligament–tibia reconstruction model (Zhu et al., 2025). Rabbits were the most commonly used experimental species and were employed in 7 studies (Zhang et al., 2023; Kim et al., 2024; Zhu et al., 2025; Chen et al., 2020; Chen et al., 2019; Bai et al., 2024; Bai et al., 2022), while rats were used in the remaining 2 studies (Ni et al., 2025; Bai et al., 2025). *In vivo* follow-up ranged from 2 to 16 weeks. The most common terminal *in vivo* time point was 12 weeks, which was used in 6 studies (Zhu et al., 2025; Bai et al., 2025; Chen et al., 2020; Chen et al., 2019; Bai et al., 2024; Bai et al., 2022), whereas 1 study extended follow-up to 16 weeks (Zhang et al., 2023). *In vitro* assessment was performed in all 9 studies and ranged from 7 to 42 days. Study characteristics are summarized in Table 1.

**TABLE 1:** Studies characteristics.

Author, year	Injury model	Animal model	Defect location and size	Experimental groups	Scaffold composition (summary)	Additive manufacturing process (summary)
Kim et al. (2024)	Full-thickness partial-width chronic rotator cuff tear	Rabbit (NZW, 2.8–3.6 kg)	5 × 5 mm supraspinatus tendon–bone	CTL/Tear/Repair/BpC/Exp (graded TBI construct)	dECM (bone + tendon) + HA + PVA + TGF-β + hASCs	3D bioprinting (core–shell, UV crosslinking)
Zhang et al. (2023)	Full-thickness rotator cuff tear (supraspinatus)	Rabbit (NZW, male, 2.5–3.0 kg)	5 × 5 mm supraspinatus tendon–bone	CTL/GBS/GBS-E (graded + dECM coating)	PCL/PLGA + HA gradient + tissue-specific dECM	Extrusion-based 3D printing + dECM post-coating
Ni et al. (2025)	Rotator cuff tear (supraspinatus)	Rat (SD, female, 0.25 kg))	6 × 4 × 1 mm supraspinatus tendon–bone footprint	Untreated/PCL/PCL + bFGF/PCL + BMSCs/PCLMF	PCL + BMSCs + bFGF	Extrusion-based 3D printing (FDM)
Zhu et al. (2025)	Full-thickness patellar ligament–tibia reconstruction	Rabbit (NZW, male 3.5 ± 0.5 kg)	4 mm diameter bone tunnel (tibial insertion)	eTi/cTi/cTi/TGF-β3	Ti6Al4V scaffold + collagen/alginate hydrogel + TGF-β3	Electron beam melting (EBM) + hydrogel infusion
Bai et al. (2025)	Rotator cuff tear (supraspinatus)	Rat (SD, male, 250–300 g)	Supraspinatus tendon–bone footprint	CTL/TSS/TCS/DTS (dual triphase)	PCL + nHA + nMgO + KGN (zone-specific layers)	Melt electrohydrodynamic (EHD) printing
Chen et al. (2020)	Rotator cuff tear (supraspinatus)	Rabbit (NZW, 1.9–2.3 kg)	10 × 5 × 0.8 mm supraspinatus tendon–bone interface	Scaffold/Scaffold + BMSCs	PLGA (50:50) ± rabbit BMSCs	3D printing (Bioplotter, melt extrusion)
Chen et al. (2019)	Rotator cuff tear (supraspinatus)	Rabbit (NZW, adult, 2.0–2.2 kg)	Supraspinatus detached from greater tuberosity	CTL/Ad-BMP-12 (BMP-12-overexpressing BM-MSCs)	PLGA (50:50) + Ad-BMP-12-transfected BM-MSCs	3D printing (Bioplotter, melt extrusion)
Bai et al. (2024)	Rotator cuff tear (supraspinatus)	Rabbit (NZW, adult male, 2.5–3.0 kg)	Supraspinatus detached from humeral footprint	CTL/S/S + D/S + R + D (SDF-1 + differentiation GFs)	PCL/PEO + chitosan NPs carrying bFGF/TGF-β/BMP-2/SDF-1	Coaxial electrohydrodynamic (EHD) printing
Bai et al. (2022)	Rotator cuff tear (supraspinatus)	Rabbit (NZW, adult male, 2.5–3.0 kg)	Supraspinatus detached from humeral footprint	CTL/Gel/Gel/μS/Gel/μS/Cells	RGD-alginate + fibrinogen hydrogel + PLGA μS (bFGF/TGF-β/BMP-2) + hUCMSCs	Custom 3D bioprinting (microfluidic printhead)

Ad-BMP-12, adenoviral vector encoding BMP-12; bFGF, basic fibroblast growth factor; BM-MSCs/BMSCs, bone marrow-derived mesenchymal stem cells; BpC, biphasic construct; CTL, control; cTi, collagen/alginate hydrogel-filled Ti scaffold; dECM, decellularized extracellular matrix; DTS, dual-triphase scaffold; EBM, electron beam melting; EHD, electrohydrodynamic; eTi, bare Ti scaffold; Exp, experimental graded TBI, construct; FDM, fused deposition modeling; GBS, graded biphasic scaffold; GBS-E, GBS, with dECM, coating; HA, hydroxyapatite; hASCs, human adipose-derived stem cells; hUCMSCs, human umbilical cord mesenchymal stem cells; KGN, kartogenin; μS, microspheres; nHA, nano-hydroxyapatite; nMgO, nano-magnesium oxide; NR, not reported; NZW, new zealand white; PCL, polycaprolactone; PCLMF, PCL, scaffold loaded with BMSCs, and bFGF; PEO, polyethylene oxide; PLGA, poly (lactic-co-glycolic acid); PVA, polyvinyl alcohol; RGD, arginine-glycine-aspartic acid; SD, Sprague-Dawley; SDF-1, stromal cell-derived factor-1; TBI, tendon–bone interface; TCS, triphase composition scaffold; TGF-β/TGF-β3, transforming growth factor-beta/beta-3; Ti6Al4V, titanium-aluminium-vanadium alloy; TSS, triphase structure scaffold; UV, ultraviolet.

### Scaffold characteristics and manufacturing approaches

All included studies evaluated three-dimensional scaffold-based constructs fabricated using additive manufacturing techniques. Extrusion-based 3D printing was used in 2 studies (Zhang et al., 2023; Ni et al., 2025), one of which specifically employed fused deposition modeling (Ni et al., 2025). Three-dimensional printing by melt extrusion using a bioplotter system was used in 2 studies (Chen et al., 2020; Chen et al., 2019). Three-dimensional bioprinting was used in 1 study (Kim et al., 2024), custom multicomponent bioprinting in 1 study (Bai et al., 2022), coaxial electrohydrodynamic printing in 1 study (Bai et al., 2024), melt electrohydrodynamic printing in 1 study (Bai et al., 2025), and electron beam melting in 1 study (Zhu et al., 2025).

Seven of the 9 studies used polymer-based constructs (Zhang et al., 2023; Ni et al., 2025; Bai et al., 2025; Chen et al., 2020; Chen et al., 2019; Bai et al., 2024; Bai et al., 2022). Polycaprolactone (PCL) was the most frequently used material and was included in 4 studies (Zhang et al., 2023; Ni et al., 2025; Bai et al., 2025; Bai et al., 2024), whereas poly (lactic-co-glycolic acid) (PLGA) was used in 4 studies (Zhang et al., 2023; Chen et al., 2020; Chen et al., 2019; Bai et al., 2022). Other scaffold components included hydroxyapatite in 2 studies (Zhang et al., 2023; Kim et al., 2024), nano-hydroxyapatite and nano-magnesium oxide in 1 study (Bai et al., 2025), polyvinyl alcohol in 1 study (Kim et al., 2024), polyethylene oxide in 1 study (Bai et al., 2024), chitosan nanoparticles in 1 study, and decellularized extracellular matrix-derived (dECM) components in 2 studies (Zhang et al., 2023; Kim et al., 2024). One study used an electron beam-melted Ti6Al4V scaffold combined with a collagen/alginate hydrogel (Zhu et al., 2025), and 1 study used a cell-laden hydrogel construct composed of RGD-alginate, fibrinogen, and growth factor-loaded PLGA microspheres (Bai et al., 2022). As for surface coatings, one study (Zhang et al., 2023) applied tissue-specific extracellular matrix coatings post-printing by low-temperature infiltration. Detailed structural properties and additive manufacturing parameters of each construct are reported in Supplementary Table S2.

### Biological augmentation

Biological augmentation was incorporated in eight studies. Cell-based strategies were used in 5 studies. Bone marrow-derived mesenchymal stem cells were used in 3 studies (Ni et al., 2025; Chen et al., 2020; Chen et al., 2019), human adipose-derived stem cells in one study (Kim et al., 2024), and human umbilical cord mesenchymal stem cells (hUCMSCs) in one study (Bai et al., 2022). Exogenous growth factors or bioactive molecules were used in 7 studies. Transforming growth factor-β (TGF-β) was used in 3 studies (Kim et al., 2024; Bai et al., 2024; Bai et al., 2022), transforming growth factor-β3 in one study (TGF-β3) (Zhu et al., 2025), basic fibroblast growth factor (bFGF) in 3 studies (Ni et al., 2025; Bai et al., 2024; Bai et al., 2022), stromal cell-derived factor-1 (SDF-1) in one study (Bai et al., 2024), bone morphogenetic protein-2 (BMP-2) in two studies (Zhu et al., 2025; Bai et al., 2022), bone morphogenetic protein-12 (BMP-12) in one study (Chen et al., 2019), and kartogenin (KGN) in one study (Bai et al., 2025). One study (Zhang et al., 2023) did not incorporate exogenous growth factors or cell-based augmentation (Zhang et al., 2023). Regarding biofunctional scaffold features, sustained or sequential release of bioactive factors was reported in five studies (Ni et al., 2025; Zhu et al., 2025; Bai et al., 2025; Bai et al., 2024; Bai et al., 2022), endogenous stem cell recruitment in one study (Bai et al., 2024), immunomodulatory activity in one study (Ni et al., 2025), and gene delivery through BMP-12-overexpressing mesenchymal stem cells in one study (Chen et al., 2019). Details of the biological augmentation strategies are summarized in Table 2.

**TABLE 2:** Augmentation strategies employed.

Author, year	Smart functionality/Structural strategy	Surface coatings	Bioactive agents and cells
Kim et al. (2024)	Spatially graded tendon–fibrocartilage–bone interface; HA gradient (tendon→bone); PVA-mediated cell alignment; core–shell bioink distribution; region-specific stiffness	Not reported	HA (osteogenic); TGF-β (tenogenic/fibrocartilage-inductive); hASCs (2 × 10^7^ cells/mL)
Zhang et al. (2023)	Triphasic scaffold with HA composition gradient increasing boneward; zone-specific stiffness; tissue-specific biochemical cues	Tissue-specific dECM (tendon-, cartilage-, bone-derived) — post-print infiltration	NR (induction media only: CTGF, TGF-β3, dexamethasone — not incorporated in scaffold)
Ni et al. (2025)	Uniform porous PCL architecture; sustained bFGF release (peak ∼2 weeks, complete by ∼4 weeks); M1→M2 immunomodulation via MAPK pathway inhibition	Not reported	bFGF (200 ng/mL, adsorbed; tenogenic/chondrogenic/osteogenic); BMSCs (1 × 10^6^ cells/mL)
Zhu et al. (2025)	Porous Ti6Al4V framework (porosity 70%, pore size 500 μm, hollow core 2 mm) + collagen/alginate hydrogel; thermo-responsive gelation at 37 °C; sustained GF release (∼21 days)	Not reported	TGF-β3 (100 ng/mL *in vitro*; 10 μg/mL *in vivo*) delivered via collagen/alginate carrier
Bai et al. (2025)	Triphasic structural + compositional gradient (DTS); zone-specific fiber spacing (100/200/400 µm for teno/chondro/osteo zones); KGN controlled release (∼50% at 42 days)	Not reported	KGN (∼100 μM; chondrogenic, sustained release); nHA (1 wt%; osteogenic); nMgO (1 wt%; bioactive/osteogenic-modulating) — all incorporated into scaffold matrix
Chen et al. (2020)	Highly porous architecture (porosity ∼98.8%); 3D geometry-induced cell alignment and collagen fiber organization	Not reported	BMSCs (rabbit-derived; cell density NR; seeded onto scaffold pre-implantation)
Chen et al. (2019)	Interconnected porous PLGA; cell-based adenoviral gene delivery (BMP-12 overexpression); tenogenic induction	Not reported	BMP-12 (via adenoviral transfection of BM-MSCs; tenogenic); BM-MSCs (1 × 10^6^ cells/scaffold; rabbit-derived)
Bai et al. (2024)	Tri-layered coaxial core–shell microfibers; layer-specific GF release (differentiation GFs in core, SDF-1 in shell); endogenous stem cell recruitment via SDF-1	Not reported	SDF-1 (shell; chemotactic, all layers); bFGF (core; tenogenic layer); TGF-β (core; chondrogenic layer); BMP-2 (core; osteogenic layer) — all via chitosan nanoparticle carriers
Bai et al. (2022)	Tri-layered living tissue construct; layer-specific GF sustained release via PLGA microspheres (∼42 days); *in situ* zonal differentiation of embedded hUCMSCs	Not reported	bFGF (tenogenic); TGF-β (chondrogenic); BMP-2 (osteogenic) — all via PLGA μS (2 µg/500 mg PLGA each); hUCMSCs (2 × 10^6^ cells/mL, all layers)

bFGF, basic fibroblast growth factor; BM-MSCs/BMSCs, bone marrow-derived mesenchymal stem cells; BMP-2, bone morphogenetic protein-2; BMP-12, bone morphogenetic protein-12; CTGF, connective tissue growth factor; dECM, decellularized extracellular matrix; DTS, dual-triphase scaffold; GF(s), growth factor(s); HA, hydroxyapatite; hASCs, human adipose-derived stem cells; hUCMSCs, human umbilical cord mesenchymal stem cells; KGN, kartogenin; μS, microspheres; nHA, nano-hydroxyapatite; nMgO, nano-magnesium oxide; NR, not reported; PCL, polycaprolactone; PLGA, poly (lactic-co-glycolic acid); PVA, polyvinyl alcohol; RGD, arginine-glycine-aspartic acid; SDF-1, stromal cell-derived factor-1; TGF-β/TGF-β3, transforming growth factor-beta/beta-3; Ti6Al4V, titanium-aluminium-vanadium alloy.

### Outcome measures

The outcome assessment was relatively consistent across the included studies and generally comprised *in vitro* biological evaluation, *in vivo* tissue-level assessment, and biomechanical testing. *In vitro* analyses mainly examined cell viability, proliferation, adhesion, morphology, and lineage-specific differentiation within the scaffold environment. *In vivo* outcomes were predominantly assessed through histological and immunohistochemical analyses, which focused on fibrocartilage formation, collagen organization, extracellular matrix deposition, and maturation of the tendon–bone interface. Two studies also used micro-computed tomography to further characterize bone regeneration. Biomechanical testing was performed in all studies, most commonly through load-to-failure and stiffness measurements, with some studies additionally reporting failure stress, tensile strength, push-out force, failure force, or energy absorption. Main outcomes are summarized in Table 3.

**TABLE 3:** Main outcomes.

Author, year	Follow-up	Sample size (N)	Key biomechanical results	Summary of main findings
Kim et al. (2024)	*In vitro*: 28 days *In vivo*: 8 weeks	*In vivo*: n = 8/group (40 total) *In vitro* assays: n = 6	Maximum load: Exp 40.2 ± 3.0 N vs. BpC 21.4 ± 2.0 N vs. Repair 7.9 ± 0.7 N (*Exp > BpC > Repair; p < 0.001*); Native CTL: 56.4 ± 9.1 N (*p < 0.001* vs*. Exp*) Stiffness: Exp 16.3 ± 2.0 N/mm vs. BpC 8.0 ± 1.2 N/mm vs. Repair 3.5 ± 0.5 N/mm (*Exp > BpC > Repair; p < 0.001*); Native CTL: 20.7 ± 2.1 N/mm (*p < 0.01* vs*. Exp*)	The biologically graded HA/TGF-β scaffold significantly improved tendon-to-bone healing, yielding the highest biomechanical performance (maximum load and stiffness), enhanced fibrocartilage formation, and superior interface maturation compared with BpC and repair groups, although values remained below native tissue
Zhang et al. (2023)	*In vitro*: 14 days *In vivo*: 8 and 16 weeks	*In vivo*: n = 12/group (36 total); biomechanics: n = 8/group/timepoint; histology: n = 4/group/timepoint *In vitro*: n = 3	Load to failure: significantly higher in GBS-E than GBS and Control at both 8 and 16 weeks (*p* < 0.05 and *p* < 0.01, respectively) Stress to failure: significantly higher in GBS-E than GBS and Control at both 8 and 16 weeks (*p* < 0.001)	The graded biomimetic scaffold coated with tissue-specific dECM significantly enhanced tendon-to-bone healing, promoting superior fibrocartilage regeneration, collagen organization, biomechanical properties, and histological scores compared with the uncoated scaffold and control groups
Ni et al. (2025)	*In vitro*: 21 days *In vivo*: 2, 4, 8 weeks	*In vivo*: n = 6/group/timepoint (90 total) *In vitro*: n = 3 Histology: n = 3/group/timepoint Biomechanics: n = 6/group/timepoint	Load to failure:: significantly higher in the PCLMF group than in all comparison groups at 2, 4, and 8 weeks; at 8 weeks, 31.3 ± 3.6 N (*p* < 0.001) Stress to failure: significantly higher in the PCLMF group than in all other groups at all postoperative time points (*p* < 0.001)	The PCL scaffold loaded with bFGF and BMSCs significantly enhanced tendon-to-bone healing by improving biomechanical strength, fibrocartilage formation, collagen organization, osteogenic differentiation, angiogenesis, and M2 macrophage polarization compared with all control groups
Zhu et al. (2025)	*In vitro*: 21 days *In vivo*: 6 and 12 weeks	*In vivo*: n = 9/group (27 total) Biomechanics: n = 3/group/timepoint *In vitro*: n = 3	Mechanical tensile strength: significantly higher in the cTi/TGF-β3 group than in eTi and cTi at both 6 and 12 weeks (vs. eTi: +14.88 ± 1.83 N and +41.40 ± 2.12 N; vs. cTi: +9.01 ± 2.11 N and +29.70 ± 1.98 N, respectively) Push-out strength: significantly higher in the cTi/TGF-β3 group than in eTi and cTi at both 6 and 12 weeks (vs. eTi: +159.3 ± 14.06 N and +199.9 ± 14.09 N; vs. cTi: +101.9 ± 10.46 N and +78.98 ± 17.90 N, respectively)	The sustained-release TGF-β3 titanium composite scaffold significantly enhanced ligament-to-bone healing by improving osseointegration, fibrocartilage regeneration, bone formation, and biomechanical fixation strength compared with titanium-only and collagen-coated scaffolds
Bai et al. (2025)	*In vitro*: 42 days *In vivo*: 6 and 12 weeks	*In vivo*: n = 6/group/timepoint (72 total) *In vitro* mechanical/degradation: N = 3	Load to failure: 27.0 ± 4.2 N at 6 weeks and 32.3 ± 2.7 N at 12 weeks, significantly higher than all control groups Stress to failure: 5.5 ± 1.0 MPa at 6 weeks and 5.2 ± 0.7 MPa at 12 weeks, significantly higher than controls	Electrohydrodynamic-printed dual-triphase microfibrous scaffolds significantly enhanced tendon-to-bone healing by promoting organized fibrocartilage formation, reducing scar tissue, improving osteochondral differentiation, and increasing the biomechanical strength of the repaired enthesis
Chen et al. (2020)	*In vivo*: 4, 8, 12 weeks	*In vivo*: n = 18/group (36 total) *In vitro*: n = 3; Histology/biomechanics: n = 6	Failure force and energy to failure were significantly improved in the PLGA + BMSCs group compared with scaffold alone at 12 weeks (*p* < 0.05). The original article presented these findings graphically without reporting numerical values (mean ± SD)	3D-printed PLGA scaffolds seeded with bone marrow-derived mesenchymal stem cells enhanced tendon-to-bone healing, increased cell infiltration, improved histological scores, promoted collagen maturation, and significantly improved the biomechanical properties of the repaired rotator cuff compared with scaffold alone
Chen et al. (2019)	*In vitro*: 7 days *In vivo*: 4, 8, 12 weeks	*In vivo*: n = 8/group (16 total) *In vitro* assays: n = 3 Biomechanical/qPCR *in vivo*: n = 6	Ultimate force to failure was significantly higher in the Ad-BMP-12 group than in the control group at 4, 8, and 12 weeks (*p* < 0.05/*p* < 0.01). Absolute biomechanical values were not reported in the original study; the native rotator cuff ultimate failure force was 97.8 ± 6.5 N	BMP-12-overexpressing BM-MSC-loaded PLGA scaffolds significantly improved tendon maturation, biomechanical strength, collagen organization, and fibrocartilage formation, resulting in enhanced tendon-to-bone healing compared with the control scaffold
Bai et al. (2024)	*In vitro*: 14 days *In vivo*: 6 and 12 weeks	*In vitro*: n = 3 *In vivo*: n = 12/group (48 total); Histology/biomechanics/immunofluorescence: n = 6/group/timepoint	Load to failure: 131.47 ± 8.95 N at 6 weeks and 150.97 ± 8.25 N at 12 weeks in the S + R + D group, significantly higher than all other groups (*P* < 0.05). No significant differences in the cross-sectional area of the rotator cuff insertion were observed among groups	The tri-layered coaxial EHD-printed core–shell scaffold with layer-specific growth factor release significantly enhanced enthesis regeneration by improving biomechanical strength, promoting organized tendon-to-bone interface reconstruction with native-like gradients, reducing inflammatory tissue, and enhancing collagen remodeling compared with the control scaffolds
Bai et al. (2022)	*In vitro*: 14 days *In vivo*: 4, 8, 12 weeks	*In vivo*: n = 6/group/timepoint (72 total); Biomechanics: n = 6/group/timepoint; *In vitro*: n = 3	The Gel/μS/Cells group showed the highest stiffness, ultimate failure load, and energy absorption at all time points. At 4 weeks, stiffness, ultimate failure load, and energy absorption were 29.32 ± 3.01 N/mm, 120.8 ± 30.5 N, and 296.24 ± 51.99 mJ, respectively. Ultimate failure load increased to 138.1 ± 22.2 N at 8 weeks and 154.3 ± 9.5 N at 12 weeks; at 12 weeks, stiffness and energy absorption reached 37.12 ± 8.38 N/mm and 453.01 ± 127.57 mJ, respectively	Bioprinted hUCMSC-laden living tissue constructs containing layer-specific growth factor-loaded microspheres promoted region-specific tenogenic, chondrogenic, and osteogenic differentiation and enhanced functional enthesis regeneration, with improved biomechanical restoration, collagen deposition and alignment, and formation of a native-like fibrocartilage gradient

Ad-BMP-12, adenoviral vector encoding BMP-12; bFGF, basic fibroblast growth factor; BM-MSCs/BMSCs, bone marrow-derived mesenchymal stem cells; BpC, biphasic construct; CTL, control; cTi, collagen/alginate hydrogel-filled Ti scaffold; dECM, decellularized extracellular matrix; DTS, dual-triphase scaffold; EHD, electrohydrodynamic; eTi, bare Ti scaffold; Exp, experimental graded TBI, construct; GBS, graded biphasic scaffold; GBS-E, GBS, with dECM, coating; GF(s), growth factor(s); HA, hydroxyapatite; hUCMSCs, human umbilical cord mesenchymal stem cells; KGN, kartogenin; μS, microspheres; NR, not reported; PCL, polycaprolactone; PCLMF, PCL, scaffold loaded with BMSCs, and bFGF; PLGA, poly (lactic-co-glycolic acid); S, scaffold only; S + D, scaffold + differentiation GFs; S + R + D, scaffold + SDF-1 + differentiation GFs; SDF-1, stromal cell-derived factor-1; TBI, tendon–bone interface; TGF-β/TGF-β3, transforming growth factor-beta/beta-3; Ti6Al4V, titanium-aluminium-vanadium alloy; TSS, triphase structure scaffold; TCS, triphase composition scaffold.

### *In vitro* outcomes

All included studies reported *in vitro* findings, and none identified relevant cytotoxic effects. Across the graded and layered constructs, spatially distinct cellular responses were reported. Hydroxyapatite-rich regions showed greater osteogenic differentiation, tendon-like regions showed higher tenogenic marker expression and fiber alignment, and intermediate regions showed fibrocartilage-related or chondrogenic differentiation (Zhang et al., 2023; Kim et al., 2024). Similar region-specific marker expression was reported in dual-triphase and tri-layered scaffolds (Zhang et al., 2023; Bai et al., 2025; Bai et al., 2024; Bai et al., 2022). This was observed in scaffolds incorporating compositional gradients, tissue-specific decellularized extracellular matrix, or layer-specific biological signals (Zhang et al., 2023; Bai et al., 2025; Bai et al., 2024; Bai et al., 2022).

Cell-based augmentation constructs generally showed increased matrix-related gene expression, and cell proliferation or lineage-specific differentiation when compared with their respective acellular or non-augmented controls (Kim et al., 2024; Ni et al., 2025; Chen et al., 2020; Chen et al., 2019; Bai et al., 2022). BMP-12-overexpressing bone marrow-derived mesenchymal stem cells showed increased tenogenic mRNA and protein expression without relevant differences in adhesion or proliferation compared with unmodified cells (Chen et al., 2019). In another study, the combination of bone marrow-derived mesenchymal stem cells and bFGF increased tenogenic, chondrogenic, and osteogenic differentiation while reducing adipogenic differentiation (Ni et al., 2025).

Controlled delivery of bioactive factors was also assessed. Sustained release of bFGF and TGF-β3 was reported for approximately 4 weeks and 21 days respectively (Ni et al., 2025; Zhu et al., 2025), while SDF-1 and layer-specific growth factors were released for up to 28 days (Bai et al., 2024). These formulations showed increased cell migration, adhesion, proliferation, or lineage-specific marker expression compared with their respective burst-release or scaffold-only controls (Ni et al., 2025; Zhu et al., 2025; Bai et al., 2024; Bai et al., 2022). Additional findings included an immunomodulatory response characterized by reduced M1 macrophage polarization and inflammatory cytokine expression, together with increased M2 polarization (Ni et al., 2025). In another study, SDF-1-mediated signaling enhanced the recruitment of endogenous mesenchymal stem cells (Bai et al., 2024).

### *In vivo* outcomes

All included studies reported *in vivo* findings, based predominantly on histological and immunohistochemical analyses, with micro-computed tomography used in selected studies. Across the included investigations, the scaffold-treated groups showed better fibrocartilage formation, collagen organization, extracellular matrix deposition, and tendon–bone interface maturation than repair alone or simpler scaffold comparators (Zhang et al., 2023; Kim et al., 2024; Ni et al., 2025; Zhu et al., 2025; Bai et al., 2025; Chen et al., 2020; Chen et al., 2019; Bai et al., 2024; Bai et al., 2022). Graded and multiphasic constructs were associated with a more distinct spatial organization of the regenerated interface. The triphasic scaffold coated with tissue-specific decellularized extracellular matrix showed greater fibrocartilage formation and collagen I and II deposition than both the uncoated scaffold and direct repair (Zhang et al., 2023). The graded or tri-layered constructs showed increased enthesis width and more organized zonal differentiation with native-like distribution of tenogenic and chondrogenic markers (Kim et al., 2024; Bai et al., 2025; Bai et al., 2024; Bai et al., 2022). Reconstruction of a four-zone enthesis and improved collagen organization were reported as early as 2 weeks in one study (Ni et al., 2025).

Cell-seeded constructs generally showed greater cellular infiltration, matrix deposition, and fibrocartilage formation than their respective acellular or non-augmented controls (Ni et al., 2025; Chen et al., 2020; Chen et al., 2019; Bai et al., 2022). BMSC-seeded PLGA scaffolds increased collagen deposition and fibrocartilage formation compared with acellular scaffolds (Chen et al., 2020). BMP-12-overexpressing BMSCs produced more advanced histological maturation of the repair tissue with a greater chondrocyte formation, and increased collagen I and III expression compared with unmodified BMSC-seeded constructs (Chen et al., 2019). The tri-layered construct incorporating human umbilical cord mesenchymal stem cells produced a wider and more organized enthesis with greater collagen alignment and deposition of collagen I, aggrecan, and osteocalcin than the acellular and growth factor-free controls (Bai et al., 2022). Implanted human umbilical cord mesenchymal stem cells remained detectable *in vivo* for up to 8 weeks (Bai et al., 2022).

Growth factor-loaded constructs also showed increased bone and fibrocartilage formation, and related expression of osteogenic or chondrogenic markers when compared with their respective controls (Ni et al., 2025; Zhu et al., 2025; Bai et al., 2024; Bai et al., 2022). The BMSC- and bFGF-loaded scaffold showed greater bone and fibrocartilage regeneration, and a better organization of the tendon–bone interface (Ni et al., 2025). The TGF-β3-loaded Ti6Al4V/hydrogel construct showed increased bone formation, and BMP-2 and collagen II expression (Zhu et al., 2025). Layer-specific delivery of SDF-1 and differentiation factors was associated with improved tissue continuity and spatial distribution of lineage-specific markers, such as SOX-9 and SCX (Bai et al., 2024). Additional findings included increased angiogenesis (Kim et al., 2024), larger collagen fibril diameter at 8 weeks (Chen et al., 2020), reduced inflammation and scar-associated tenascin-C expression, and increased aggrecan deposition (Bai et al., 2025; Bai et al., 2022).

### Biomechanical outcomes

All included studies performed biomechanical testing, although the reported endpoints varied: these included load to failure, stiffness, failure stress, tensile strength, push-out force, and energy absorption. Across the included investigations, the experimental constructs showed higher biomechanical values than their respective simpler controls (Zhang et al., 2023; Kim et al., 2024; Ni et al., 2025; Zhu et al., 2025; Bai et al., 2025; Chen et al., 2020; Chen et al., 2019; Bai et al., 2024; Bai et al., 2022).

Among studies evaluating scaffold architecture, graded and multiphasic constructs showed higher load-bearing capacity than simpler comparators. The graded scaffold evaluated by Kim et al. (Kim et al., 2024) showed higher load to failure and stiffness than the biphasic and untreated controls, while the dECM-coated triphasic scaffold investigated by Zhang et al. (2023) showed higher ultimate load and ultimate stress than both the uncoated scaffold and direct repair at 8 and 16 weeks. Similarly, the dual-triphase construct reported by Bai et al. (2025) achieved higher failure load and failure stress than scaffolds incorporating only structural or compositional gradients.

Cell-seeded constructs also showed improved biomechanical outcomes compared with their respective controls. BMSC seeding increased failure force and energy absorption compared with an acellular PLGA scaffold (Chen et al., 2020), whereas BMP-12-overexpressing BMSCs produced higher ultimate force to failure than unmodified BMSC-seeded scaffolds at all evaluated time points (Chen et al., 2019). The PCL scaffold containing BMSCs and bFGF showed higher maximum load and stiffness than all comparator groups (Ni et al., 2025), while the tri-layered construct containing human umbilical cord mesenchymal stem cells showed the highest ultimate failure load, stiffness, and energy absorption among the tested groups (Bai et al., 2022).

Growth factor-loaded constructs showed similar differences. The TGF-β3-loaded Ti6Al4V/hydrogel scaffold achieved higher tensile strength and push-out force than both the hydrogel-only and bare titanium scaffolds (Zhu et al., 2025). The tri-layered scaffold incorporating SDF-1 and layer-specific differentiation factors showed the highest ultimate failure load among its comparator groups at both 6 and 12 weeks (Bai et al., 2024).

Native-tissue benchmarks were not consistently reported. When available, the regenerated interfaces showed higher biomechanical values than comparator groups but remained below those of the intact enthesis (Kim et al., 2024; Bai et al., 2022). In Bai et al. (Bai et al., 2022), the cell-laden construct approached native ultimate failure load by 12 weeks, whereas Kim et al. (Kim et al., 2024) reported that load to failure and stiffness remained lower than native values.

### Risk of bias

The methodological quality assessment performed using SYRCLE’s Risk of Bias tool for animal studies showed an overall unclear risk of bias across the included studies (Table 4). This was mainly attributable to insufficient reporting of methodological items. In contrast, baseline characteristics were consistently judged as low risk, indicating adequate comparability between experimental groups at study entry. Incomplete outcome data were generally assessed as low risk in several studies, whereas selective outcome reporting was most often judged as unclear because of limited detail regarding predefined outcomes and reporting protocols. Overall, although no study showed a uniformly high risk of bias, the frequent lack of methodological detail reduced confidence in the internal validity of the available preclinical evidence.

**TABLE 4:** Risk of bias was assessed using SYRCLE’s Risk of Bias tool for animal studies.

Study	Sequence generation	Baseline characteristics	Allocation concealment	Random housing	Blinding (Caregivers/Investigators)	Random outcome assessment	Blinding (outcome assessor)	Incomplete outcome data	Selective outcome reporting	Other bias	Overall judgment
Kim et al. (2024)	Unclear	Low	Unclear	High	Unclear	Low	Low	Low	Unclear	Unclear	UNCLEAR: insufficient reporting of sequence generation, allocation concealment, and blinding procedures.
Zhang et al. (2023)	Unclear	Low	Unclear	Unclear	Unclear	Unclear	Low	Low	Unclear	Low	UNCLEAR: insufficient reporting of key data, despite adequate reporting of baseline characteristics, sample size, and blinded histological assessment.
Ni et al. (2025)	Unclear	Low	Unclear	Low	Unclear	Unclear	Unclear	Low	Unclear	Low	UNCLEAR: insufficient reporting of sequence generation, allocation concealment, and blinding procedures.
Zhu et al. (2025)	Unclear	Low	Unclear	Unclear	Unclear	Unclear	Unclear	Unclear	Unclear	Unclear	UNCLEAR: randomization methods, allocation concealment, and blinding procedures were not adequately reported.
Bai et al. (2025)	Unclear	Low	Unclear	Unclear	Unclear	Unclear	Unclear	Low	Unclear	Unclear	UNCLEAR: incomplete reporting of randomization, allocation concealment, and blinding.
Chen et al. (2020)	Unclear	Low	Unclear	Unclear	Unclear	Unclear	Unclear	Unclear	Unclear	Unclear	UNCLEAR: insufficient reporting of randomization procedures, allocation concealment, and blinding.
Chen et al. (2019)	Unclear	Low	Unclear	Unclear	Unclear	Unclear	Unclear	Unclear	Unclear	Unclear	UNCLEAR lack of reporting on randomization methods, allocation concealment, and blinding procedures.
Bai et al. (2024)	Unclear	Low	Unclear	Unclear	Unclear	Unclear	Unclear	Low	Unclear	Unclear	UNCLEAR: insufficient reporting of allocation concealment, blinding procedures, random housing, and random outcome assessment
Bai et al. (2022)	Unclear	Low	Unclear	Unclear	Unclear	Unclear	Low	Low	Unclear	Unclear	UNCLEAR: insufficient reporting of randomization procedures, allocation concealment, and blinding

## Discussion

The present review synthesized the available preclinical evidence on additively manufactured 3D scaffold-based strategies for enthesis regeneration. To date, this is the first systematic review specifically focused on original preclinical studies investigating additively manufactured 3D scaffold-based constructs for enthesis regeneration. Although limited to animal studies, the included investigations showed a consistent pattern: these constructs improved biological, histological, and biomechanical healing at the tendon–bone interface compared with their respective controls. Across the reviewed studies, the most recurrent advantages were enhanced lineage-specific differentiation *in vitro*, greater fibrocartilage formation and collagen organization *in vivo*, and superior mechanical performance during biomechanical testing. At the same time, the current evidence also indicates that regeneration of a fully native enthesis remains difficult to achieve, as even the most advanced constructs generally improved repair without completely reproducing the structure and function of the normal insertion.

One of the most relevant findings of this review is the central role of scaffold architecture. Several studies showed that constructs designed to reproduce the spatial complexity of the native enthesis yielded better outcomes than simpler comparators. Kim et al. (2024) demonstrated that a graded scaffold promoted region-specific differentiation and improved interface maturation compared with a biphasic construct. Similarly, Zhang et al. (2023) found that a triphasic graded scaffold enhanced spatial differentiation and mechanical performance, particularly when further functionalized with tissue-specific decellularized extracellular matrix. Bai et al. (2025) also reported that a dual-triphase scaffold combining structural and compositional gradients outperformed designs incorporating only one of these features. Taken together, these findings suggest that the regenerative benefit depends not only on the presence of a physical support, but also on their ability to reproduce the zonal transitions that characterize the native tendon–bone interface. The *in vitro* findings further support this interpretation. Across the included studies, scaffold-based systems were generally cytocompatible and capable of supporting cell proliferation, adhesion, and differentiation. More importantly, the most advanced constructs were able to guide region-specific cellular behavior. Kim et al. (2024), Zhang et al. (2023), Bai et al. (2024), and Bai et al. (2022) all showed that graded or layered scaffolds promoted spatially distinct responses. These data therefore support the concept that scaffold design can actively direct cellular organization and differentiation, which is essential for more physiological interface reconstruction. This is particularly relevant in enthesis regeneration, as functional healing depends on re-establishing the graded tendon–fibrocartilage–bone transition of the native insertion rather than forming disorganized scar tissue. Enthesis architecture determines how load is transferred across the interface. Indeed, the native enthesis is a specialized transition with graded structure from tendon to bone: this relies on opposing gradients of collagen fiber alignment and mineral content to manage stress transfer (Peniche Silva et al., 2022). This gradient reduces stress concentrations and allows forces to be distributed more smoothly (Apostolakos et al., 2014). Graded interfaces allow for a progressive transition in stiffness, so strain can be partitioned more gradually rather than localizing at one point (Boys et al., 2019). Second, they improve load transfer by distributing forces across a broader interfacial region, which reduces peak stress and increases toughness (Rossetti et al., 2017). Plus, the native enthesis also uses hierarchical fiber organization and interdigitation, which further disperses stress and limits failure at the soft–hard junction (Thomopoulos et al., 2006). During joint movement, the enthesis must continuously withstand severe tensile, compressive, and shear forces (Thomopoulos et al., 2006). Functional healing requires restoration of this complex interface rather than simple fibrous repair, which is mechanically inferior and associated with a higher risk of failure and retear (Derwin et al., 2018; Bousso et al., 2024; Wildemann and Klatte, 2012; Prabhath et al., 2018). Indeed, Kim et al. (2024) reported higher load-to-failure and stiffness in the graded scaffold group than in control groups; while Zhang et al. (2023) observed that the decellularized extracellular matrix-coated triphasic graded scaffold achieved higher ultimate load and ultimate stress than controls.

All included studies reported improvements in at least one mechanical parameter in the experimental groups. In general, constructs that combined complex architecture with biologic augmentation achieved the best mechanical outcomes. This was evident in the studies by Ni et al. (2025), Bai et al. (2024), and Bai et al. (2022), all of which showed superior mechanical performance relative to multiple comparators. At the same time, the available data also highlight a persistent translational gap. When native tissue was used as a benchmark, regenerated interfaces generally remained biomechanically inferior (Kim et al., 2024), even when substantial improvements over controls were observed. Therefore, while current scaffold designs appear capable of enhancing repair quality, they do not yet reliably restore native mechanical competence. This could be also a time-scale issue, since most preclinical studies assess healing at relatively early time points (Apostolakos et al., 2014). Indeed, histologically immature regenerated tissue could fail to match the hierarchical organization of native tissue, which was shown to distribute load and reduce stress concentration (Bousso et al., 2024).

Another major theme emerging from the reviewed studies is the role of biological augmentation. Eight of the 9 studies incorporated cells, growth factors, or other bioactive signals, and these biologically augmented constructs generally showed more favorable histological and biomechanical outcomes than their respective comparators. Cell-based approaches were investigated in 5 studies (Kim et al., 2024; Ni et al., 2025; Chen et al., 2020; Chen et al., 2019; Bai et al., 2022) and were associated with greater matrix deposition and improved fibrocartilage formation. Moreover, cell-based approaches were associated with an increased biomechanical response with respect to comparator groups. In particular, the studies by Chen et al. (Chen et al., 2020; Chen et al., 2019) showed that bone marrow-derived mesenchymal stem cell seeding, especially when combined with BMP-12 overexpression, improved histological maturation and increased ultimate force to failure. Bai et al. (Bai et al., 2022) further demonstrated that a tri-layered cell-laden construct with human umbilical cord mesenchymal stem cells produced biomechanical values approaching those of native tissue by 12 weeks. Overall, these findings suggest that scaffold architecture and cell-based augmentation may act synergistically, with the scaffold providing spatial guidance and the seeded cells enhancing matrix synthesis and tissue remodeling. Growth factor delivery represented another consistent strategy. Transforming growth factor-β, transforming growth factor-β3, basic fibroblast growth factor, BMP-2, BMP-12, stromal cell-derived factor-1, and kartogenin were incorporated across the included studies (Kim et al., 2024; Ni et al., 2025; Zhu et al., 2025; Bai et al., 2025; Chen et al., 2019; Bai et al., 2024; Bai et al., 2022). These approaches were generally associated with enhanced lineage-specific differentiation *in vitro* and improved fibrocartilage regeneration and mechanical performance *in vivo*. Importantly, several studies did not simply add bioactive molecules to the scaffold, but engineered systems for sustained or region-specific release. Ni et al. (Ni et al., 2025) showed that sustained bFGF release was associated with enhanced osteogenesis, chondrogenesis, tenogenesis, and reduced inflammatory signaling. Zhu et al. (Zhu et al., 2025) similarly found that a TGF-β3-loaded composite scaffold improved osteochondral regeneration and fixation strength. Bai et al. (Bai et al., 2024; Bai et al., 2022) incorporated layer-specific growth factor delivery into tri-layered constructs and reported favorable structural and mechanical outcomes. These results suggest that the mode of factor delivery may be as important as the factor itself, since controlled release may better reproduce the temporal and spatial complexity of the healing enthesis. Enthesis healing is a coordinated multistage process involving an early inflammatory phase, followed by progenitor-cell recruitment; these cells undergo controlled region-specific differentiation, fibrocartilage formation and mineralization, and remodeling under mechanical load (Lu and Thomopoulos, 2013; Samorezov and Alsberg, 2015; Lei et al., 2021). Failure to regulate these events spatially and temporally tends to result in scar-mediated repair rather than regeneration of the native tendon–fibrocartilage–bone interface (Lu and Thomopoulos, 2013; Samorezov and Alsberg, 2015; Lei et al., 2021). The reviewed studies also suggest that the role of 3D scaffolds is evolving beyond passive structural replacement: some constructs were specifically designed to modulate the biological environment of healing. Ni et al. (Ni et al., 2025) reported reduced inflammatory cytokine expression together with a shift from M1 toward M2 macrophage polarization, indicating an immunomodulatory effect. Bai et al. (Bai et al., 2024) used stromal cell-derived factor-1 to enhance endogenous stem cell recruitment, while Chen et al. (Chen et al., 2019) used BMP-12-overexpressing mesenchymal stem cells to potentiate tenogenic signaling. Scaffold-based strategies should therefore be designed to support tissue formation mechanically, and at the same time to provide biological cues to regulate inflammation, recruit reparative cells, and direct differentiation across the tendon–bone transition. However, spatio-temporal control of bioactive cues could represent an issue for enthesis scaffolds, since these need to simultaneously deliver tenogenic cues on one end to promote tendon differentiation, and osteogenic or chondrogenic factors on the other end to promote bone integration (Micalizzi et al., 2023). In case these molecules diffuse improperly, it may lead to chaotic and unorganized tissue growth (Micalizzi et al., 2023). Another issue observed in scaffold-mediated repair is ectopic or undesired endochondral ossification, which is mineralization spreading directly into the tendon zone causing localized pain and severe tendon weakness (Peniche Silva et al., 2022). However, none of the included studies reported cases of ectopic ossification.

From a material standpoint, most studies relied on polymer-based systems, particularly polycaprolactone and poly (lactic-co-glycolic acid) (Zhang et al., 2023; Ni et al., 2025; Bai et al., 2025; Chen et al., 2020; Chen et al., 2019; Bai et al., 2024; Bai et al., 2022). These materials likely remain attractive because they are printable and compatible with a wide range of biologic modifications, while at the same time mechanically stable (Dwivedi et al., 2020; Li et al., 2022). However, their limitations should also be acknowledged: PCL degrades slowly and is relatively hydrophobic, which may limit cell interaction unless modified, whereas PLGA degrades more rapidly and can generate acidic by-products that may affect the local microenvironment (Xie et al., 2025; Song et al., 2025). The included studies also showed that polymeric scaffolds alone may not be sufficient to support the full complexity of enthesis regeneration. Mineral phases were used to promote osteogenic differentiation and mineralized interface formation (Zhang et al., 2023; Kim et al., 2024; Bai et al., 2025). However, these components may increase scaffold brittleness and, if not spatially controlled, may compromise the gradual transition required for effective tendon–bone integration (Wu et al., 2020). In the included studies, tissue-specific extracellular matrix components were incorporated to improve lineage-specific responses (Zhang et al., 2023; Kim et al., 2024). Zhu et al. (Zhu et al., 2025) explored a different hybrid strategy by combining a metallic Ti6Al4V scaffold with a collagen/alginate hydrogel, thereby integrating mechanical stability with a more favorable biologic microenvironment. Overall, these findings suggest that material selection should be considered in functional rather than purely compositional terms, with each component contributing specific structural or biological properties to the regenerative process (Gonzalez-Fernandez et al., 2019; Patel et al., 2018). Beyond ensuring mechanical stability and printability, the selected materials should enable controlled presentation of bioactive cues and guide region-specific differentiation (Gonzalez-Fernandez et al., 2019; Patel et al., 2018). Moreover, the scaffold must maintain its mechanical strength long enough to bear physiological loads while native tissue grows into it, yet degrade progressively to allow gradual load transfer to the remodeling collagen (Chen et al., 2025). Many synthetic materials suffer from quick biodegradation (losing strength before the tissue matures) or low bioactivity, which fails to attract the necessary host cells to jumpstart regeneration (Li et al., 2022). In the included studies, these scaffolds were cytocompatible, and no overt cytotoxic effects were reported. Several studies also showed sustained regenerative activity over time, with progressive tissue formation and prolonged biological effects consistent with continued functional cue delivery (Ni et al., 2025; Zhu et al., 2025). However, scaffold degradation kinetics were only rarely investigated in detail. As a result, although these materials appeared capable of supporting ongoing regeneration, it remains unclear whether their degradation profiles were optimally synchronized with tissue maturation and gradual load transfer to the newly formed matrix.

In summary, *in vivo* findings across the 9 studies were broadly consistent: improved fibrocartilage formation and collagen alignment, more mature extracellular matrix deposition, and better interface organization were reported in nearly all experimental groups compared with controls. Some studies also reported more specific features of advanced healing, such as tidemark development (Zhang et al., 2023), restoration of the four-layer enthesis organization (Ni et al., 2025), greater enthesis width (Bai et al., 2022), and reduced scar-associated tenascin-C expression (Bai et al., 2025; Bai et al., 2022). These observations are important because histological appearance at the tendon–bone interface is closely linked to long-term load transmission. In this context, the reviewed studies suggest that 3D scaffold-based approaches can shift healing away from fibrous scar formation and toward a more organized insertion-like tissue. Nevertheless, this regenerative effect was variable in magnitude, and no study demonstrated complete recreation of a fully native enthesis. Another important issue concerns clinical translation, as many of the most effective constructs combined multiphasic architecture, seeded cells and multiple bioactive factors, or release systems (Ni et al., 2025; Zhu et al., 2025; Bai et al., 2024; Bai et al., 2022). Although these strategies produced favorable experimental results, their translation to clinical practice remains challenging. In particular, advanced scaffold fabrication may be technically demanding and difficult to standardize at scale, while cell-based or growth factor-loaded constructs raise additional regulatory and cost-related concerns. Further issues include sterilization without loss of biological activity and reproducibility of manufacturing. As a result, the gap between experimental efficacy and clinical applicability remains substantial.

Several limitations of the current evidence base should be considered. First, all included studies were preclinical animal investigations and therefore provided only level V evidence. Most were conducted in rabbit models, with 2 studies performed in rats (Ni et al., 2025; Bai et al., 2025), and none included large animal models. As a result, the ability of these findings to predict clinical performance in humans remains uncertain. Second, the duration of follow-up was relatively short. Most studies terminated at 12 weeks, and only 1 extended to 16 weeks (Zhang et al., 2023). This limits assessment of long-term remodeling, durability of the regenerated interface, scaffold degradation behavior, and maintenance of mechanical function over time. Third, there was substantial methodological heterogeneity across studies, including differences in animal model, scaffold composition, manufacturing technique, biologic augmentation, comparator groups, outcome measures, and biomechanical testing protocols. This heterogeneity precluded any quantitative synthesis and makes it difficult to isolate the effect of individual scaffold components. Another limitation of the available evidence is the generally unclear methodological quality of the included studies. The risk-of-bias assessment showed frequent insufficient reporting of sequence generation, allocation concealment, random housing, and blinding procedures, which reduces confidence in the internal validity of the reported findings. An important issue concerns clinical translation. Many of the most effective constructs were also among the most complex, combining multiphasic architecture, seeded cells, multiple bioactive factors, or engineered release systems (Ni et al., 2025; Zhu et al., 2025; Bai et al., 2024; Bai et al., 2022). Although these strategies produced favorable experimental results, their complexity may limit scalability, reproducibility, regulatory approval, and cost-effectiveness. In particular, cell-based and growth factor-loaded constructs may be difficult to implement in routine clinical practice. Future development will likely need to balance biologic sophistication with translational feasibility, identifying which scaffold features are truly essential for regeneration and which add complexity without clear practical benefit.

Future research should therefore pursue both mechanistic refinement and translational simplification. Longer-term studies are needed to determine whether early histological and biomechanical gains are sustained over time. Large animal models should also be considered to better reproduce the dimensions and mechanical environment of the human enthesis. In parallel, more standardized reporting of histological, molecular, and biomechanical outcomes would improve comparability across studies. Finally, scaffold development should aim to identify clinically feasible designs that preserve the key advantages observed in the current literature, particularly spatially organized architecture and controlled biological signaling, while reducing manufacturing and regulatory burden.

## Conclusion

The available evidence from short-term animal studies suggests that additively manufactured 3D scaffold-based strategies may enhance enthesis healing, especially when biomimetic scaffold architecture is combined with biological augmentation. However, the regenerated interfaces generally remained inferior to native tissue and no clinical evidence is currently available. Moreover, the existing preclinical literature is limited by short follow-up and substantial heterogeneity. Accordingly, these findings should be interpreted cautiously, and further long-term preclinical and clinical studies are required before any conclusions on clinical applicability can be drawn.

## Data Availability

The original contributions presented in the study are included in the article/Supplementary Material, further inquiries can be directed to the corresponding author.

## Glossary

3D: three-dimensional
ACAN: aggrecan
Ad-BMP-12: adenoviral vector encoding bone morphogenetic protein-12
ALP: alkaline phosphatase
ARS: Alizarin Red S staining
BdECM: bone-derived decellularized extracellular matrix
BGN: biglycan
BM-MSCs/BMSCs: bone marrow-derived mesenchymal stem cells
BMD: bone mineral density
BMP-2: bone morphogenetic protein-2
BMP-12: bone morphogenetic protein-12 (=GDF-7)
BpC: non-graded (biphasic) construct
bFGF: basic fibroblast growth factor
BV/TV: bone volume/total volume
CCK-8: Cell Counting Kit-8
CL: cardiolipin
COL1/COL1A1: collagen type I/type I collagen alpha-1
COL2/COL2A1: collagen type II/type II collagen alpha-1
COL3/COL3A1: collagen type III
COL10A1: collagen type X alpha-1
Ctl/CTL: control group
cTi: collagen/alginate hydrogel-filled titanium scaffold
CTGF: connective tissue growth factor
DAPI: 4′,6-diamidino-2-phenylindole (nuclear stain)
DCM: dichloromethane
dECM: decellularized extracellular matrix
DTS: dual-triphase scaffold (combined structural + compositional gradient)
EBM: electron beam melting
ECM: extracellular matrix
EHD: electrohydrodynamic (printing)
ELISA: enzyme-linked immunosorbent assay
eTi: bare (uncoated) titanium scaffold
Exp: experimental graded TBI construct
FU: follow-up
GAG: glycosaminoglycan
GBS: graded biphasic scaffold
GBS-E: graded biphasic scaffold with dECM coating
GDF-7: growth and differentiation factor-7 (=BMP-12)
Gel: hydrogel construct without microspheres
Gel/μS: hydrogel construct with layer-specific GF-loaded microspheres (acellular)
Gel/μS/Cells: cell-laden hydrogel construct with layer-specific GF-loaded microspheres
GF(s): growth factor(s)
H&E: haematoxylin and eosin staining
HA: hydroxyapatite
hASCs: human adipose-derived stem cells
HFIP: hexafluoroisopropanol
HLA-ABC: human leukocyte antigen ABC
hUCMSCs: human umbilical cord mesenchymal stem cells
IF: immunofluorescence
IHC: immunohistochemistry
IL-1β: interleukin-1 beta
IL-6: interleukin-6
IOD: integrated optical density
KGN: kartogenin
LDH: lactate dehydrogenase assay
LOE: level of evidence
MAPK: mitogen-activated protein kinase
MOD: mean optical density
MT: Masson’s trichrome staining
MTT: 3-(4,5-dimethylthiazol-2-yl)-2,5-diphenyltetrazolium bromide assay
micro-CT: micro-computed tomography
nHA: nano-hydroxyapatite
nMgO: nano-magnesium oxide
OCN: osteocalcin
OPN: osteopontin
ORO: Oil Red O staining
PC: phosphatidylcholine
PCL: polycaprolactone
PECAM-1: platelet endothelial cell adhesion molecule-1 (CD31)
PEO: polyethylene oxide
PI: phosphatidylinositol
PLGA: poly(lactic-co-glycolic acid)
PCLMF: PCL scaffold loaded with BMSCs and bFGF
PPARγ: peroxisome proliferator-activated receptor gamma
PS: phosphatidylserine
PVA: polyvinyl alcohol
qRT-PCR/RT-qPCR: quantitative reverse transcription polymerase chain reaction
RGD: arginine-glycine-aspartic acid (integrin-binding cell adhesion peptide)
RUNX2: runt-related transcription factor 2
S: scaffold only (no GFs)
S + D: scaffold with differentiation GFs only (no SDF-1)
S + R + D: scaffold with recruitment GF (SDF-1) and differentiation GFs
SCX: scleraxis
SDF-1: stromal cell-derived factor-1 (CXCL12)
SEM: scanning electron microscopy
SO: Safranin O staining
SOFG: Safranin O–Fast Green staining
SOX9: SRY-box transcription factor 9
SR: Sirius Red staining
Tb.N: trabecular number
Tb.Sp: trabecular separation
Tb.Th: trabecular thickness
TBI: tendon–bone interface
TCS: triphase composition scaffold
TdECM: tendon-derived decellularized extracellular matrix
TEM: transmission electron microscopy
TGF-β/TGF-β3: transforming growth factor-beta/beta-3
TG: triglyceride
Ti6Al4V: titanium-aluminium-vanadium alloy
TNC: tenascin-C
TNMD: tenomodulin
TNF-α: tumour necrosis factor-alpha
TPP: sodium tripolyphosphate
TSS: triphase structure scaffold
UPLC-MS/MS: ultra-performance liquid chromatography tandem mass spectrometry
UV: ultraviolet
VEGF: vascular endothelial growth factor
WB: Western blot
μS: microspheres
