# Supplementary material for: 3D-printed scaffold-based strategies for enthesis regeneration: a systematic review of the literature
Source: Front Bioeng Biotechnol. 2026 Aug 31;14:1876882. doi: 10.3389/fbioe.2026.1876882 (PMC13572595; doi:10.3389/fbioe.2026.1876882)
Supplement: Supplementary file 1 [file Table1.docx]

| **Database** | **Search Strategy** |
| --- | --- |
| PubMed | ("enthesis" OR "tendon-bone interface" OR "bone-tendon interface" OR "osteotendinous junction") AND ("scaffold" OR "scaffolds" OR "biomaterial" OR "biomaterials" OR "tissue engineering" OR "3D printing" OR "bioprinting" OR "additive manufacturing" OR "multiphasic" OR "gradient" OR "functionally graded") AND ("healing" OR "regeneration" OR "repair" OR "fibrocartilage" OR "histology" OR "histological" OR "osteogenesis" OR "chondrogenesis" OR "tenogenesis" OR "biomechanical" OR "mechanical testing" OR "ultimate load" OR "stiffness" OR "failure stress" OR "energy to failure" OR "pull-out test" OR "tensile test") |
| Embase | ('enthesis':ti,ab,kw OR 'tendon-bone interface':ti,ab,kw OR 'bone-tendon interface':ti,ab,kw OR 'osteotendinous junction':ti,ab,kw) AND ('scaffold':ti,ab,kw OR 'scaffolds':ti,ab,kw OR 'biomaterial':ti,ab,kw OR 'biomaterials':ti,ab,kw OR 'tissue engineering':ti,ab,kw OR '3D printing':ti,ab,kw OR 'bioprinting':ti,ab,kw OR 'additive manufacturing':ti,ab,kw OR 'multiphasic':ti,ab,kw OR 'gradient':ti,ab,kw OR 'functionally graded':ti,ab,kw) AND ('healing':ti,ab,kw OR 'regeneration':ti,ab,kw OR 'repair':ti,ab,kw OR 'fibrocartilage':ti,ab,kw OR 'histology':ti,ab,kw OR 'histological':ti,ab,kw OR 'osteogenesis':ti,ab,kw OR 'chondrogenesis':ti,ab,kw OR 'tenogenesis':ti,ab,kw OR 'biomechanical':ti,ab,kw OR 'mechanical testing':ti,ab,kw OR 'ultimate load':ti,ab,kw OR 'stiffness':ti,ab,kw OR 'failure stress':ti,ab,kw OR 'energy to failure':ti,ab,kw OR 'pull-out test':ti,ab,kw OR 'tensile test':ti,ab,kw) |
| Word of Science Core Collection | TS=("enthesis" OR "tendon-bone interface" OR "bone-tendon interface" OR "osteotendinous junction") AND TS=("scaffold" OR "scaffolds" OR "biomaterial" OR "biomaterials" OR "tissue engineering" OR "3D printing" OR "bioprinting" OR "additive manufacturing" OR "multiphasic" OR "gradient" OR "functionally graded") AND TS=("healing" OR "regeneration" OR "repair" OR "fibrocartilage" OR "histology" OR "histological" OR "osteogenesis" OR "chondrogenesis" OR "tenogenesis" OR "biomechanical" OR "mechanical testing" OR "ultimate load" OR "stiffness" OR "failure stress" OR "energy to failure" OR "pull-out test" OR "tensile test") |
