# Supplementary material for: 3D-printed scaffold-based strategies for enthesis regeneration: a systematic review of the literature
Source: Front Bioeng Biotechnol. 2026 Aug 31;14:1876882. doi: 10.3389/fbioe.2026.1876882 (PMC13572595; doi:10.3389/fbioe.2026.1876882)
Supplement: Supplementary file 2 [file Table2.docx]

| **Author, Year** | **Detailed Structural Properties** | **Additive Manufacturing Parameters** | **Bioactive Agent Delivery Details** |
| --- | --- | --- | --- |
| Kim et al., 2025 [17] | Strut diameter ~300 µm; HA gradient distribution (tendon→bone); aligned tendon-zone fibers; region-specific stiffness; ↑ compressive strength with increasing HA content | Core–shell nozzle; pneumatic extrusion; in situ UV crosslinking (Irgacure 2959 photoinitiator); spatially controlled flow rates per zone | PVA (0.1 g/mL) for cell alignment; Irgacure 2959 photoinitiator for crosslinking; TGF-β (10 ng/mL) and HA (~20 wt%) incorporated in bioinks; hASCs (2×10⁷ cells/mL) |
| Zhang et al., 2023 [11] | Pore size ~150 µm (tendon and transition zones), ~300 µm (bone zone); fully interconnected porous scaffold; dimensions ~10×10×1 mm; Young's modulus gradient increasing tendon→bone; HA-driven composition-dependent stiffness | 400 µm nozzle; pneumatic dispensing; multi-step zone-specific ink printing; DCM solvent; post-print dECM coating via low-temperature infiltration | Tissue-specific dECM (tendon-, cartilage-, bone-derived) applied post-printing by low-temperature infiltration in DCM solvent; no exogenous GFs incorporated in scaffold (induction media used in vitro only) |
| Ni et al., 2025 [18] | Pore size ~300 µm; scaffold dimensions ~6×4×1 mm; stable mechanical properties after cell loading; elastic modulus from stress–strain curves; complete degradation ~4 wk in vivo | LivPrint3D system; controlled nozzle temperature, speed, and layer thickness; fiber diameter and pore size ~300 µm | bFGF (200 ng/mL) adsorbed onto scaffold surface; sustained release profile (peak ~2 wk, complete ~4 wk) vs burst release in controls; BMSCs (1×10⁶ cells/mL) seeded pre-implantation |
| Zhu et al., 2025 [19] | Porosity 70%; pore size ~500 µm; strut ~300 µm; hollow core diameter 2 mm; homogeneous hydrogel filling confirmed; stable structural integrity; ↑ mechanical integration over time in vivo | EBM for Ti6Al4V scaffold fabrication; post-processing; collagen (0.3% w/v) and alginate (5% w/v) hydrogel infusion; in situ thermal gelation at 37°C | TGF-β3: 100 ng/mL in vitro; 10 µg/mL in vivo; delivered via collagen/alginate hydrogel carrier; sustained release ~97% cumulative at 21 d; thermo-responsive sol→gel transition at 37°C |
| Bai et al., 2025 [20] | Fiber width ~16.3 ± 1.8 µm; layer spacing ~98/203/408 µm (teno/chondro/osteo zones); layer thickness ~109 µm; good interlayer adhesion (no delamination); uniform particle distribution; degradation ~44% (lipase) / ~63% (NaOH) at 21 d | Melt EHD printing; layer-by-layer deposition; controlled fiber spacing and architecture per zone | KGN (~100 µM): ~50% release at 42 d; nHA (1 wt%) and nMgO (1 wt%) incorporated into scaffold matrix; tensile properties stable after drug loading |
| Chen et al., 2020 [21] | Pore size ~861 ± 22 µm; porosity ~98.8%; highly interconnected architecture; scaffold degradation: ~50% remaining at 4 wk, ~30% at 8 wk, ~5% at 12 wk | Bioplotter (EnvisionTEC); melt extrusion; pneumatic dispensing | BMSCs (rabbit-derived; cell density NR) seeded onto scaffold pre-implantation; no exogenous GFs incorporated |
| Chen et al., 2019 [22] | Porosity 79.3 ± 5.4%; pore size 256.3 ± 17.6 µm; scaffold diameter 15 mm, height 1 mm; fully interconnected porous structure; mass loss (PBS): ~25% at 4 wk, ~43% at 8 wk, ~76% at 12 wk | Bioplotter (EnvisionTEC); 27-gauge metal needle; 135°C; ~600 kPa dispensing pressure; XY head speed 80 mm/min | BMP-12 delivered via adenoviral transfection (Ad-BMP-12) of BM-MSCs pre-seeding; BM-MSCs (1×10⁶ cells/scaffold) seeded post-transfection |
| Bai et al., 2024 [23] | Core–shell fiber width 24.3 ± 6.3 µm; total scaffold thickness 314.5 ± 16.0 µm; pore size ~81.5/173.3/388.9 µm (teno/chondro/osteo zones); tensile modulus 8.56→4.82 MPa (after PBS); tensile strength 667.3→451.4 kPa (after PBS); scaffold degraded by 12 wk in vivo; hierarchical nanofibrous surface after PBS (PEO leaching) | Coaxial EHD printing; HFIP solvent (evaporation-based); 2 kV applied voltage; nozzle-to-collector distance 2.5 mm; layer-by-layer zone-specific deposition; fiber spacings 100/200/400 µm; independent core/shell syringe pumps | Chitosan nanoparticles (ionotropic gelation with TPP) as GF carriers; SDF-1 in shell (all layers, chemotactic); bFGF/TGF-β/BMP-2 in core (layer-specific); GF release: rapid burst in first 48 h, sustained over 28 d; SDF-1 release slightly faster than core GFs |
| Bai et al., 2022 [24] | Filament width 233.7 ± 44.0 µm; filament spacing 500 µm; total construct thickness 279.3–300 µm; tensile modulus: Gel 478.0→Gel/μS 154.2→Gel/μS/Cells 42.9 kPa; tensile strength: Gel 312.0→Gel/μS/Cells 159.8 kPa; ultimate tensile strain Gel/μS/Cells 444.6 ± 75.2%; toughness 0.46 ± 0.10 ×10⁻⁶ J/m³; degradation ~80% mass at 1 d → ~17% at 18 d (collagenase) | Custom 3D bioprinting system with microfluidic-based multicomponent printhead; pneumatic extrusion; agarose/CaCl₂ collecting substrate for instant ionic crosslinking; flow rate 800 µL/h; stage speed 30 mm/s; CaCl₂/thrombin secondary crosslinking | PLGA μS (75:25, Mw=70,000; particle size 1.4 ± 0.9 µm; encapsulation efficiency ~58.2%); bFGF/TGF-β/BMP-2 (2 µg/500 mg PLGA each); GF release: ~30% at 2 d, ~40% at 14 d (μS) vs burst ~40% at 2 h (without μS); hUCMSCs (2×10⁶ cells/mL) in all layer bioinks; cells detectable in vivo up to 8 wk |
| bFGF, basic fibroblast growth factor; BM-MSCs/BMSCs, bone marrow-derived mesenchymal stem cells; BMP-2, bone morphogenetic protein-2; BMP-12, bone morphogenetic protein-12; DCM, dichloromethane; dECM, decellularized extracellular matrix; EBM, electron beam melting; EHD, electrohydrodynamic; GF(s), growth factor(s); HA, hydroxyapatite; HFIP, hexafluoroisopropanol; hUCMSCs, human umbilical cord mesenchymal stem cells; KGN, kartogenin; μS, microspheres; nHA, nano-hydroxyapatite; nMgO, nano-magnesium oxide; NR, not reported; PBS, phosphate-buffered saline; PCL, polycaprolactone; PEO, polyethylene oxide; PLGA, poly(lactic-co-glycolic acid); PVA, polyvinyl alcohol; SDF-1, stromal cell-derived factor-1; TGF-β/TGF-β3, transforming growth factor-beta/beta-3; Ti6Al4V, titanium-aluminium-vanadium alloy; TPP, sodium tripolyphosphate; UV, ultraviolet. | | | |
